# Toward integrative genomics study of genetic resistance to *Salmonella* and *Campylobacter* intestinal colonization in fowl

**DOI:** 10.3389/fgene.2012.00261

**Published:** 2012-12-14

**Authors:** Fanny Calenge, Catherine Beaumont

**Affiliations:** ^1^INRA, UMR1313 Génétique Animale et Biologie IntégrativeJouy-en-Josas cedex, France; ^2^INRA, UR083 Unité de Recherches AvicolesNouzilly, France

**Keywords:** *Salmonella*, *Campylobacter*, chicken, QTL, genetic architecture, intestinal colonization, carrier-state, candidate gene

## Abstract

*Salmonella enterica* serotypes Enteritidis and Typhimurium and *Campylobacter jejuni* are responsible for most cases of food poisoning in Europe. These bacteria do not cause severe disease symptoms in chicken, but they are easily propagated by symptomless chicken carriers which cannot be easily isolated. This animal tolerance is detrimental to food safety. In this particular case, increasing animal's resistance is not sufficient, since some animals considered as resistant are able to carry bacteria during several weeks without displaying disease symptoms. We review studies aimed at evaluating the resistance of chicken to *Salmonella* and *Campylobacter* intestinal colonization, either a few days or several weeks after infection. While studies of the genetic control of *Campylobacter* colonization are only beginning, mostly due to technical difficulties in infection protocols, genetic studies of *Salmonella* colonization have been conducted for now more than 20 years. They have initially reported an estimation of the genetic parameters associated with resistance to *Salmonella* colonization and are now aimed at identifying the genomic regions controlling variation of this trait in experimental lines and commercial populations. With the advent of high-throughput genomics, we are closer than ever to identify the true genes controlling resistance to *Enterobacteria* colonization in chicken. The comparison of genes involved in early resistance to intestinal colonization with genes controlling resistance to bacteria persistence several weeks after infection (i.e., carrier-state) should soon highlight the differences between the molecular mechanisms underlying those two distinct phenotypes. It will also be highly interesting to compare the genes or genomic regions controlling *Campylobacter* and *Salmonella*, in order to evaluate the feasibility of a selection conducted on both bacteria simultaneously.

## Introduction

According to the most recent EFSA report about food-borne outbreaks in Europe, *Campylobacter*, followed by *Salmonella*, are responsible for most of the reported isolated cases of food-borne diseases, while outbreaks are mostly due to *Salmonella* (EFSA, 2012). These Gram negative *Enterobacteria* live in the intestinal tract of livestock animals (poultry, pigs, and bovine). Bacteria infecting human consumers derive mainly from contaminated avian products, i.e., broiler meat and raw eggs. The main *Salmonella* serotype responsible for human illness, i.e., *Salmonella enterica* serotype Enteritidis, is able to infect broiler chickens or laying hens without causing disease symptoms. Human illness due to *Campylobacter* is mainly due to the species *Campylobacter jejuni*, which is similarly responsible for a silent chicken infection. This animal's ability to carry zoonotic bacteria without showing disease symptoms causes a silent propagation of bacteria in poultry stocks due to the impossibility to isolate contaminated animals. These bacteria are not a threat to animal health but are detrimental to food safety.

Prophylactic measures taken by European countries to clear poultry flocks from *Salmonella* firstly focused on breeder flocks. To prevent vertical transmission, those flocks were systematically checked for absence of contamination by strains of major impact on human health and culled in case of contamination. These procedures have been shown to be efficient (EFSA, 2010) and are now practiced in most flocks. However, they are not sufficient to completely eliminate *Salmonella* Enteritidis and are only efficient in case of vertical propagation, which occurs only for some serotypes of *Salmonella* but not for *Campylobacter*. Genetic selection could be a valuable alternative. The aim of selection in this case would not be to obtain healthy animals since most animals show no disease symptoms, but rather to select more resistant animals with reduced intestinal colonization. In this particular case, animals show an extreme form of tolerance since bacteria colonization is not detrimental to the host health and performance. Simulation studies have shown that using animals more resistant to *Salmonella* intestinal persistence (defined as carrier-state) in combination with vaccination is indeed efficient to reduce *Salmonella* propagation in laying hen stocks (Prévost et al., 2006, 2008).

As previously reviewed (Calenge et al., 2010), two types of studies related to *Salmonella* intestinal colonization are currently conducted, according to the delay considered after experimental infection, i.e., either a few days or several weeks. Resistance to early *Salmonella* intestinal colonization has been mainly studied at Iowa State University (USA) by a candidate gene approach and at the Institute for Animal Health (IAH, Compton, UK), first by comparison of different chicken lines and more recently by looking for genomic regions controlling intestinal colonization. A similar approach has been undertaken at the National Institute for Agronomical Research (INRA, France) in order to study resistance to bacteria persistence several weeks after infection, defined as resistance to carrier-state.

Resistance to *Campylobacter* intestinal colonization in poultry has been more rarely studied, probably due to technical difficulties for cultivating these anaerobic bacteria and performing reproducible infection tests. The emergence of sanitary concerns about the presence of these bacteria on animal products, especially on broiler carcasses (EFSA, 2012), has reinforced the scientific interest for these bacteria. Only a few studies have already been published, mentioning differences in response to *Campylobacter* infection according to the chicken line tested (Stern et al., 1990; Boyd et al., 2005), which opens the way to genetic selection and to more in-depth genetics studies.

In this paper, we present a review of results obtained on the genetic control of resistance to intestinal colonization by *Campylobacter* and *Salmonella* in fowl. We then discuss the possibility of a partially common genetic control of: (1) resistance to early colonization and to persistence on the one hand, (2) resistance to *Campylobacter* and to *Salmonella* on the other hand. We eventually discuss the scientific opportunities offered by the existence of multiple infection models for the study of *Salmonella* infection, and the necessity of an integrative genomics approach to better understand the genetic control of resistance to *Enterobacteria* carrier-state.

## Genetic control of resistance to *salmonella* intestinal colonization in chicken

In the 1980s, researchers and breeders began to take an interest in the serotypes responsible for human cases of salmonellosis, i.e., *S*. Enteritidis and *S*. Typhimurium, while previous scientific studies had been focusing on species specific serotypes (*S*. Gallinarum, *S*. Pullorum) causing acute salmonellosis in chickens (Wigley, 2004; Calenge et al., 2010). Different infections models have been used to evaluate resistance to intestinal colonization by these serotypes (Calenge et al., 2010). The main differences between these models are the age at which experimental infections are carried out (either young chicks/hatchlings or adult laying hens), the age at which the level of colonization is measured (a few days or several weeks p.i.) and the way intestinal colonization is measured (cecal load or fecal shedding). Studying very young chicks is essential since commercial broilers are often infected at a very young age. On the other hand, bacteria excretion is a great concern for laying hens when hens reach the laying peak, since bacteria can easily contaminate egg shells. To evaluate intestinal colonization, bacteria are counted in ceca, which is a reservoir for intestinal bacteria, or in feces. The level of intestinal colonization measured a few days after infection evaluates the *Salmonella* shedding potential of each bird immediately after infection. Nevertheless, it does not allow an estimation of persistent shedding, which can only be evaluated several weeks after infection.

A series of publications investigated the role of candidate genes otherwise known for their role in immunity in the observed variability of cecal load, 1 week after infection of 1-day old chicks (Calenge et al., 2010). Several genes, namely *CD28*, *IAP1*, *TGF*-β_2,3,4_, *Gal*_11,12,13_, *TRAIL*, *IL*_−2,10_, *PSAP*, *SLC11A1*, *IGL*, *CASP1*, *iNOS*, *PIGR*, and *MAPKAPK12* were actually associated with variation for cecal load of *S*. Enteritidis (Kaiser et al., 1998, 2002; Kaiser and Lamont, 2001, 2002; Lamont et al., 2002; Liu et al., 2002, 2003; Kramer et al., 2003; Malek and Lamont, 2003; Malek et al., 2004; Hasenstein et al., 2006). The effects of the candidate genes *SLC11A1* and *TLR4* have been largely studied in several experimental populations (Calenge et al., 2010). Nevertheless none of those genes had a major effect. To complete these studies, the effects of these genes should be studied in other populations in order to evaluate their stability and importance in the control of *Salmonella* intestinal colonization. This has been done for *SLC11A1* and *TLR4* in several independent studies, which failed to identify major and stable effects of these genes.

To study *S*. Enteritidis persistence, two models of infection were developed at INRA, differing in the age at which animals are infected: at 1 week of age (Duchet-Suchaux et al., 1995, 1997) or at the laying peak (Protais et al., 1996). Conditions were chosen with which all animals are carrying bacteria shortly after infection but are able to get rid of them in a few weeks (Duchet-Suchaux et al., 1995; Protais et al., 1996). Measures are made several weeks after infection in order to evaluate the animal's ability to completely clear pathogenic *Salmonella* from its digestive tract. After studies conducted to estimate the heritability of resistance to carrier-state (Girard-Santosuosso et al., 1998, 2002), a divergent selection experiment was conducted on commercial laying hens (Beaumont et al., 2009). Interestingly, this experiment showed that genetic resistance at a young age was negatively correlated to adult resistance. In other words, some genes contributing to carrier-state resistance at a young age have an antagonistic effect on adult animals. This could be related to the immaturity of the immune system in chicks, which implies that some of the genes controlling resistance to carrier-state could be involved in the immune response.

In order to identify the genomic regions controlling resistance to *S*. Enteritidis intestinal carrier-state, QTL analyses experiments were then carried out using experimental White Leghorn Inbred lines. These lines had been shown to display different levels of resistance to different serotypes of *Salmonella* (Bumstead and Barrow, 1988, 1993; Bumstead et al., 1991). The first analysis was a selective genotyping approach using a F2 progeny derived from parental inbred lines N and 6_1_, conducted only on animals displaying extreme phenotypes (Tilquin et al., 2005). It was followed by a confirmation study after genotyping the whole F2 progeny (Calenge et al., 2009). Both studies used microsatellite genotypings. The two most significant QTLs were identified and confirmed on chromosomes 2 and 16. Interestingly, the QTL on chromosome 16 is located on the Major Histocompatibility Complex, so that one of the genes belonging to this complex is probably the actual gene at the QTL. A following analysis was performed with a more complete and denser genome scan using 480 highly informative SNP markers and a higher number of animals. It led to several QTLs on previously uncovered microchromosomes but failed to confirm the major QTL on chromosome 2, while the effect of the QTL located on the MHC on chromosome 16 could not be confirmed due to the absence of segregating SNP markers in this genome region (Calenge et al., 2011). To test the influence of the detection method on the QTL identified, an additional analysis was performed using maximum likelihood, whereas previous studies used linear regression. With the maximum likelihood method developed in the MapQTL software, the possibility of gene segregation within the parental lines could be taken into account. Intriguingly, although phenotypic and genotypic data were identical, QTL were completely different (Tran et al., 2012). This apparent discrepancy is probably a consequence of the different hypotheses underlying both calculation methods, which have a greater impact on QTL with weak effects. In addition, dominance effects could not be taken into account with the maximum likelihood method used, so that all QTL with a strong dominant effect could not be detected. In parallel, a similar QTL analysis of *Salmonella* early intestinal colonization has been carried out at IAH using a distinct infection model in which animals were evaluated a short time after infection (Fife et al., 2011). Interestingly, two of the four QTL detected are located close to QTL controlling *S*. Enteritidis persistence, so that it can be speculated that these QTL have pleiotropic effects both on *S*. Typhimurium early colonization and on *S*. Enteritidis persistence (Tran et al., 2012).

On the whole, these candidate gene and QTL analysis studies show a complex control of *Salmonella* intestinal colonization in laying hens, with many QTL or candidate genes having weak effects varying according to animal's age, parental lines, and also QTL detection method. The detection of one QTL on the MHC and the influence if animal's age on QTL detection lead us to the hypothesis that some of the genes controlling carrier-state are involved in the immune response. This would be coherent with the assumption that a better resistance to *Salmonella* early colonization is one of the mechanisms leading to better resistance to carrier-state. At this stage, although some of the QTLs identified have been validated in commercial lines (Calenge et al., 2009), marker assisted selection is not possible because of the small effects of QTLs and of their large confidence intervals.

## A common genetic control for resistance to *salmonella* and *campylobacter* carrier-state?

*Campylobacter* and *Salmonella* are both Gram negative *Enterobacteria* living in the host intestine, silently carried by chickens and causing gastro-intestinal disease in humans. For these reasons, both are a concern for food safety rather than for animal health. These similarities naturally lead to the conclusion that the genetic control of carrier-state could be at least partly common for these bacteria. Only a few studies have been published about the genetic control of *Campylobacter* resistance. A first study in 1990 showed genetic differences between caecal loads of three commercial broiler lines (Stern et al., 1990). It was followed by a comparison of several White Leghorn inbred layer lines, which showed significant differences in the number of bacteria in the caeca or cloaca between the lines studied (Boyd et al., 2005). Another study demonstrated differences in *C. jejuni* cecal colonization between two different broiler lines (Li et al., 2008). Interestingly, the same inbred layer lines N and 6 that display different levels of resistance to *Salmonella* carrier-state showed different levels of resistance to *Campylobacter* colonization, which strengthens the hypothesis of a common genetic control of both bacteria (Boyd et al., 2005). At INRA a first, preliminary comparison of different chicken lines for their resistance level to *C. jejuni* carrier-state was conducted. It included the N and 6 lines, with a different infection model developed at ANSES (Ploufragan, France). Nevertheless it did not reveal significant differences in carrier-state levels between the lines studied, with the exception of Fayoumi which showed a lower level of *C. jejuni* carrier-state. This shows the great influence of the infection protocol on the results observed. A more recent gene expression study of the local cecal response to *Campylobacter* colonization mentions two broiler lines differing for their susceptibility to *Campylobacter* (Li et al., 2008, 2010). This study identified distinct transcriptional profiles between both lines, with genes identified for the first time in avian infection studies (Li et al., 2010). These results strengthen the hypothesis of a genetic control of resistance to *Campylobacter* and also tends to favor the hypothesis of genetic control specific to this bacterial species.

A recent QTL analysis of *Campylobacter* colonization was performed in a progeny derived from lines N and 6 by using the infection protocol developed by Boyd et al. (2005). Four QTL with locations independent from those of the QTL for *Salmonella* colonization were identified in a similar progeny, i.e., a backcross population [6 × N] × N (Kaiser, 2010). The author concluded to the absence of common resistance genes for both bacteria, which is not so surprising, when citing the author, considering that *Campylobacter* and *Salmonella* infections differ in their physiopathology and in the innate immune responses involved (Shaughnessy et al., 2009; Kaiser, 2010). Nevertheless, *Campylobacter* QTL locations were compared only with those of QTL for resistance to *Salmonella* colonization, and not with those of QTL for *Salmonella* carrier-state. It appears that three of the QTL identified, on chromosomes 7, 11, and 27, co-localize (i.e., their confidence intervals overlap) with QTL for resistance to carrier-state (Tilquin et al., 2005; Calenge et al., 2009, 2011). Therefore, although there is probably no unique genetic resistance control for both bacteria, some genes could be common when considering carrier-state and not only early colonization. It would be much interesting to know ultimately in which part of the immune resistance mechanisms those common genes are involved: innate or acquired resistance, tolerance mechanisms, etc. In short, chicken line comparison studies available are apparently contradictory, probably due to differences in the infection protocols used, while comparison of QTL analyses points to QTL co-locations between resistance to *Salmonella* carrier-state and resistance to *Campylobacter* colonization. These results show the absence of an obvious common genetic determinism but do not discard the possibility of a few common genes. Further research is needed to better understand the genetic architecture of resistance to *Campylobacter* carrier-state, with much attention paid to the infection protocol used, since different protocols can lead to opposite conclusions.

Future studies should also take into account the host intestinal microbiota, since recent research conducted both on human and livestock demonstrates the previously underestimated impact of this microbiota on the host ability to mount an immune response and to control pathogens (Kosiewicz et al., 2011). Interactions between gut microbiota and immune system have already been demonstrated in chicken (Brisbin et al., 2008). The role of microbiota in the establishment of an immune response after *S*. Enteritidis has already been questioned (Crhanova et al., 2011) and the microbiota response to a challenge by *C. jejuni* has been studied (Qu et al., 2008). In order to colonize host intestines, pathogenic *Enterobacteria* must overcome the resistance mediated by the gut microbiota and the innate immune system. While some studies conclude to the absence of effect of *Salmonella* or *Campylobacter* colonization on host microbiota composition (Qu et al., 2008; Nordentoft et al., 2011), others mention effects of *S*. Enteritidis colonization on the gut immune response when compared to normal microbiota (Crhanova et al., 2011). If microbiota composition does not change following *Campylobacter* or *Salmonella* colonization, which should be confirmed in other studies, it does not preclude any change in functional interactions between microbiota and host immune response. This area of research is worth being further explored. If feasible, influencing microbiota composition through host genetic selection or nutrition could be an indirect way to limit tolerance to intestinal pathogens. A recent study using mouse advanced intercross lines (AIL) has demonstrated the role of host genetics control in shaping individual microbiome diversity (Benson et al., 2010). Authors define a core measurable microbiota which variations are under host genetic control. It would be particularly interesting to know if host genes determine microbiota composition in chicken and if those genes have an indirect impact on pathogenic *Enterobacteria* colonization and carrier-state.

## Many infection protocols and phenotypes to study *salmonella* infection: weakness or strength?

Since QTL for *Salmonella* carrier-state identified are relatively unstable according to many parameters (chicken age, calculation method), to strengthen our results we turned to other studies conducted using other *Salmonella* infection protocols. The many differences in the infection protocols used to study *Salmonella* resistance or carrier-state render comparisons of results difficult, since protocols differ in many ways and it is impossible to decipher which condition exactly led to different results (Calenge et al., 2010). Nevertheless, the reliability and interest of QTL are strengthened when QTL detected using two different infection protocols and co-localizing. Co-location can provide some hints on the possible way of action of co-localizing QTLs, although with the great size of QTL confidence intervals, these considerations are speculative and have to be confirmed by more in-depth studies. This is what led us to underline the interest of QTLs identified on chromosomes 2 and 3, which were also identified in independent studies of *Salmonella* Typhimurium early colonization in the 6_1_ and 15I White Leghorn inbred lines (Fife et al., 2011). Using SNP markers located close to these QTL (Fife et al., 2011), QTL detection was slightly improved (Tran et al., 2012). One or several genes controlling early colonization to *Salmonella* could thus very well be involved in the control of *Salmonella* carrier-state. Another example illustrating the interest of comparing QTL locations from independent studies interested in different phenotypes is the study of Redmond et al. (2011), compared to results of Fife et al. (2009). It appears that SNP markers associated with heterophil function were identified very close to the gene *SIVA1*, candidate for the major QTL *SAL1*, involved in the control of splenic *S*. Typhimurium load (Mariani et al., 2001; Fife et al., 2009). Since *SIVA1* is a likely regulator of heterophil function, its co-location with SNP markers involved in heterophil function strengthens the plausibility of its causal role for the *SAL1* major QTL (Redmond et al., 2011). The other interest of this study is the great precision of the phenotype assessed, which gives access to possible gene functions. A finer phenotyping of resistance taking into account all levels of host reaction to invading pathogens, i.e., from disease symptoms to the molecules and cells involved in innate or adaptive immune response, through the composition of host gut microbiota and the intestinal immune response, should be considered as an interesting strategy to characterize the functions of QTLs and strengthen plausible positional candidate genes. These examples of QTL co-location show that the existence of many different *Salmonella* infection protocols can be seen as strength to characterize QTL functions rather than a weakness preventing comparisons. Ideally, QTL or gene locations should be compared on the same animal material. When truly causal genes will be eventually identified, the existence of multiple infection models will enable researchers to understand the genetic origin of differences between early colonization and carrier-state, but also between *Salmonella* and *Campylobacter* infection.

## Toward causal genes identification: necessity of an integrative approach

The identification of the causal genes underlying QTL for resistance to carrier-state and to colonization would be a great progress toward a better understanding of the mechanisms differentiating *Enterobacteria* true resistance and carrier-state. Are causal genes involved in the innate or adaptive immune response or key regulators genes controlling several metabolic pathways? Are they directly or indirectly responsible for a shift in gut microbiota composition or involved in mechanisms circumventing or escaping immune resistance mechanisms? Those questions will be answered only when causal genes will be identified. Until now at least, classical QTL analyses have found their limits. QTL confidence intervals are too vast to reasonably point to one or several candidate genes, with the only exception of the major QTL *SAL1* (Mariani et al., 2001), which phenotypic effects were important enough to allow classical genetics studies to identify only a few candidate genes (Fife et al., 2009). Two striking candidate genes were proposed for this QTL: *SIVA1*, coding for the CD27-binding protein Siva and *AKT1*, coding for the RAC-alpha serine/threonine protein kinase homolog (Fife et al., 2009). More generally, before choosing candidate genes, QTL locations need to be refined. AIL are a material of choice to reach this purpose (Darvasi and Soller, 1995). They have already been successfully used in chicken to refine QTL for body weight (Besnier et al., 2011) or QTL affecting resistance to Marek's disease (Heifetz et al., 2009). Thanks to the advent of high-throughput genotyping, their high rate of recombinations can now easily be exploited to fine map QTLs. Interestingly, an independent study confirmed *SIVA1* as most probable candidate for *SAL1* by looking for SNP markers associated with heterophil function in AIL of chicken (Redmond et al., 2011). The latter study identified SNP markers associated with heterophils extra-cellular trap (HET) production, thus indicating a possible role for *SIVA1* as a regulator of HET production (Redmond et al., 2011). This study well demonstrates the interest of coupling QTL fine-mapping strategies with high density genotyping to reduce QTL confidence intervals. It is probable that this strategy was successful with *SAL1* due to the importance of its effect on splenic *Salmonella* colonization. It can be questioned whether the exploitation of AIL will be fruitful for QTL with much weaker effect: QTL confidence intervals will probably be refined, but not to a single or even a few candidate(s), unless very striking candidate genes appear.

To reach causative genes at QTLs, it seems relevant to conduct an integrative approach by leading several types of analyses simultaneously, in order both to cross-validate QTL locations and to characterize their functions. Comparing QTL locations identified in independent studies is interesting. Although it does not lead to causal genes, it can give hints regarding their function by linking different phenotypes. This was done to confirm the probable role of *SAL1* in regulating heterophil function (Redmond et al., 2011). Candidate gene approaches, taken alone, are not sufficient to explain the totality of phenotype variations, but when candidate genes co-localize with QTL they become even more interesting. This is why the involvement of functional candidate genes, annotated to be involved in the immune response or differentially expressed between parental lines, could be more systematically investigated. Many expression studies before/after challenge with pathogenic *Enterobacteria* have been conducted, sometimes with different chicken lines, and their results could be better taken into account (Calenge et al., 2010). Before the availability of the chicken genome sequence, candidate gene approaches have successfully been conducted in chicken to study resistance to *Salmonella* carrier-state. The roles of the genes *SLC11A1* (previously named *NRAMP1*) and *TLR4* have been studied in several chicken lines (Girard-Santosuosso et al., 2002; Lamont et al., 2002; Beaumont et al., 2003; Kramer et al., 2003; Leveque et al., 2003; Calenge et al., 2009). Many gene related to the immune response have also been the object of focused studies (Lamont et al., 2002; Liu et al., 2002; Kramer et al., 2003; Malek and Lamont, 2003; Malek et al., 2004; Hasenstein et al., 2006; Hasenstein and Lamont, 2007; Ghebremicael et al., 2008). This candidate gene approach only led to the detection of slight effects, which is coherent with what was observed for QTL analyses, with many QTL of small to medium effect. From these two different approaches it appears that resistance to *Salmonella* carrier-state is apparently controlled by several genes of small effect, probably varying according to chicken breed, chicken age, parameters related to the infection protocol used (i.e., inoculum dose, time post infection, etc.). This is most probably the case for resistance to other *Enterobacteria*. Furthermore, a recent study demonstrates the role of epigenetic regulation of TLR gene expression in the resistance to *S*. Enteritidis colonization (Gou et al., 2012). It would be much interesting to know whether those epigenetic modifications are under host genetic control.

## Conclusion

Integrative studies allowed by the advent of high-throughput genomics should soon lead to causal genes for *Enterobacteria* intestinal colonization in chicken. Nevertheless, even in the most favorable case in which we know several causal genes, genetic selection will be complicated by their weak to medium effect and by their instability according to chicken line, age and environment. In addition, intestinal colonization by *Campylobacter* and *Salmonella* are probably not controlled by the same genes. In this context, could genomic selection be considered as the obvious solution for commercial selection of chicken more resistant to bacteria intestinal colonization? It seems promising, since SNP markers causing variation for the trait considered are directly selected within the selection stock without looking for causal genes (Goddard et al., 2010), thus preventing the need for checking QTL or gene stability according to many parameters. Indeed, a first study gave interesting results for SNP-assisted selection for resistance to *Salmonella* carrier-state in laying hens (Legarra et al., 2011). Nevertheless, for the time being many obstacles stand in the way of genomic selection for disease resistance in chicken. One of them is the necessity to challenge, before any application and repeatedly during the selection process, a very high number (several thousands) of animals belonging to the reference population. This seems hardly feasible for the study of *Salmonella* or *Campylobacter* intestinal colonization, which implies to count bacteria in caeca or spleen, even if both diseases have a high economic and social impact and can thus be considered as traits of interest for commercial application (Davies et al., 2009). Alternatively, integrative genomics approaches combined with recent dramatic advances in genotyping costs and efficiency should soon lead to the identification of causal genes at the QTL, thus precluding the need for recurrent test of the association of causal genes with disease resistance and leading to much more accurate and reliable genomic assessment.

### Conflict of interest statement

The authors declare that the research was conducted in the absence of any commercial or financial relationships that could be construed as a potential conflict of interest.
